# 2-[4,5-Bis(ethyl­sulfan­yl)-1,3-dithiol-2-yl­idene]-5-(4-methoxy­phen­yl)-5*H*-1,3-dithiolo[4,5-*c*]pyrrole

**DOI:** 10.1107/S1600536809047424

**Published:** 2009-11-14

**Authors:** Feng-Shou Leng, Bao Li, Bing-Zhu Yin, Li-Xin Wu

**Affiliations:** a Key Laboratory of Organism Functional Factors of Changbai Mountain, Yanbian University, Ministry of Education, Yanji 133002, People’s Republic of China; b State Key Laboratory of Supramolecular Structure and Materials, College of Chemistry, Jilin University, Changchun 130012, People’s Republic of China

## Abstract

The asymmetric unit of the title compound, C_19_H_19_NOS_6_, contains two independent mol­ecules with different conformations of the ethyl groups. The dihedral angles between the pyrrole and benzene rings are 14.19 (14) and 16.29 (17)° in the two molecules. In the absence of short inter­molecular contacts, in the crystal, the mol­ecules are packed with their long axes parallel to [10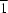].

## Related literature

For general background to the use of pyrrolo-tetra­thia­fulvalene derivatives as building blocks in supra­molecular and materials chemistry, see Becher *et al.* (2004 ▶). For details of the synthesis, see Yin *et al.* (2004 ▶). 
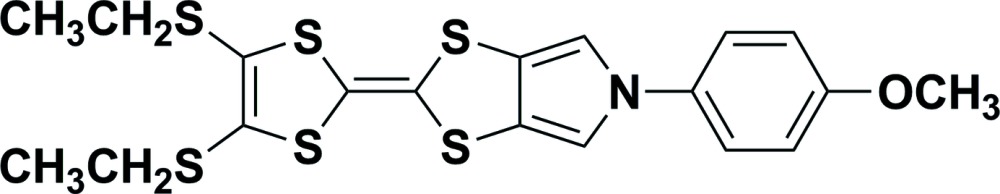


## Experimental

### 

#### Crystal data

C_19_H_19_NOS_6_
*M* *_r_* = 469.71
Triclinic, 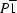

*a* = 11.974 (2) Å
*b* = 12.121 (2) Å
*c* = 16.762 (3) Å
α = 73.90 (3)°
β = 85.53 (3)°
γ = 65.43 (3)°
*V* = 2123.8 (7) Å^3^
*Z* = 4
Mo *K*α radiation
μ = 0.65 mm^−1^
*T* = 291 K
0.14 × 0.13 × 0.12 mm

#### Data collection

Rigaku R-AXIS RAPID diffractometer
Absorption correction: multi-scan (*ABSCOR*; Higashi, 1995 ▶) *T*_min_ = 0.914, *T*_max_ = 0.926
21057 measured reflections
9633 independent reflections
6504 reflections with *I* > 2σ(*I*)
*R*_int_ = 0.033

#### Refinement

*R*[*F* ^2^ > 2σ(*F* ^2^)] = 0.041
*wR*(*F* ^2^) = 0.117
*S* = 1.01
9633 reflections
493 parameters
H-atom parameters constrained
Δρ_max_ = 0.31 e Å^−3^
Δρ_min_ = −0.27 e Å^−3^

### 

Data collection: *RAPID-AUTO* (Rigaku, 1998 ▶); cell refinement: *RAPID-AUTO*; data reduction: *CrystalStructure* (Rigaku/MSC, 2002 ▶); program(s) used to solve structure: *SHELXS97* (Sheldrick, 2008 ▶); program(s) used to refine structure: *SHELXL97* (Sheldrick, 2008 ▶); molecular graphics: *PLATON* (Spek, 2009 ▶); software used to prepare material for publication: *SHELXL97*.

## Supplementary Material

Crystal structure: contains datablocks global, I. DOI: 10.1107/S1600536809047424/cv2638sup1.cif

Structure factors: contains datablocks I. DOI: 10.1107/S1600536809047424/cv2638Isup2.hkl

Additional supplementary materials:  crystallographic information; 3D view; checkCIF report

## Figures and Tables

**Fig. 1.**
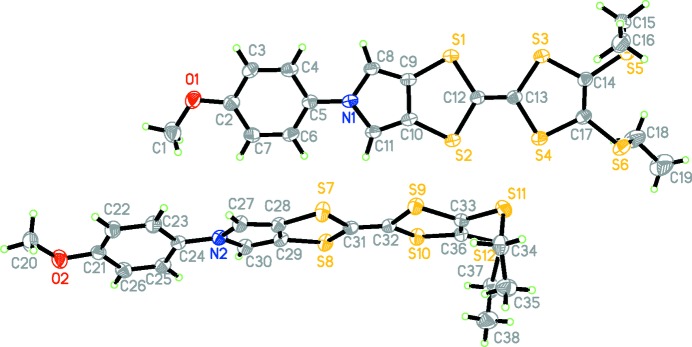
Two independent molecules of the title compound, with the atom numbering and 30% probalility displacement ellipsoids.

## supplementary crystallographic information

### Comment

Pyrrolo-tetrathiafulvalene derivatives, an important class of electron-donors, are versatile building blocks in supramolecular and materials chemistry (Becher *et al.*, 2004). Recently, we have reported the synthesis and eletron donor properties of the title compound, *N*-(4-methoxyphenyl)pyrrolo[3,4-*d*] tetrathiafulvalene (Yin *et al.*, 2004). In this paper, we report its crystal structure.

The asymmetric unit of the title compound contains two independent molecules (Fig. 1), M1 and M2, respectively. The tetrathiafulvalene and benzene ring are nearly coplanar in both M1 and M2 molecules. Conformations of ethyl groups in M1 and M2 are different: two ethyls in M1 lie in the different side of the plane, while at the same side in M2. In the absence of short intermolecular contacts, the crystal packing is featured by packing of enlongated molecules parallel to direction [10-1].

### Experimental

The title compound was prepared through cross-coupling reaction of 4,5-bis(ethylthio)-1,3-dithiole-2-thione with *N*-(4-methoxyphenyl)-(1,3)-dithiolo[4,5-*c*]pyrrole-2-one (Yin *et al.*, 2004). Single crystals for X-ray diffraction were pepared by slow evaporation a mixture of dichloromethane and petroleum at room temperature.

### Refinement

C-bound H-atoms were placed in calculated positions (C—H 0.93-0.97 Å), and were included in the refinement in the riding model, with *U*_iso_(H) = 1.5 or 1.2 *U*_eq_(C).

### Crystal data

**Table:**

C_19_H_19_NOS_6_	*Z* = 4
*M**_r_* = 469.71	*F*(000) = 976
Triclinic, *P*1	*D*_x_ = 1.469 Mg m^−^^3^
Hall symbol: -P 1	Mo *K*α radiation, λ = 0.71073 Å
*a* = 11.974 (2) Å	Cell parameters from 14243 reflections
*b* = 12.121 (2) Å	θ = 3.1–27.5°
*c* = 16.762 (3) Å	µ = 0.65 mm^−^^1^
α = 73.90 (3)°	*T* = 291 K
β = 85.53 (3)°	Block, yellow
γ = 65.43 (3)°	0.14 × 0.13 × 0.12 mm
*V* = 2123.8 (7) Å^3^	

### Data collection

**Table:**

Rigaku R-AXIS RAPID diffractometer	9633 independent reflections
Radiation source: fine-focus sealed tube	6504 reflections with *I* > 2σ(*I*)
graphite	*R*_int_ = 0.033
ω scans	θ_max_ = 27.5°, θ_min_ = 3.1°
Absorption correction: multi-scan (*ABSCOR*; Higashi, 1995)	*h* = −15→13
*T*_min_ = 0.914, *T*_max_ = 0.926	*k* = −15→15
21057 measured reflections	*l* = −21→21

### Refinement

**Table:**

Refinement on *F*^2^	Primary atom site location: structure-invariant direct methods
Least-squares matrix: full	Secondary atom site location: difference Fourier map
*R*[*F*^2^ > 2σ(*F*^2^)] = 0.041	Hydrogen site location: inferred from neighbouring sites
*wR*(*F*^2^) = 0.117	H-atom parameters constrained
*S* = 1.01	*w* = 1/[σ^2^(*F*_o_^2^) + (0.0602*P*)^2^] where *P* = (*F*_o_^2^ + 2*F*_c_^2^)/3
9633 reflections	(Δ/σ)_max_ = 0.001
493 parameters	Δρ_max_ = 0.31 e Å^−^^3^
0 restraints	Δρ_min_ = −0.26 e Å^−^^3^

### Special details

**Table:**

Experimental. (See detailed section in the paper)
Geometry. All e.s.d.'s (except the e.s.d. in the dihedral angle between two l.s. planes) are estimated using the full covariance matrix. The cell e.s.d.'s are taken into account individually in the estimation of e.s.d.'s in distances, angles and torsion angles; correlations between e.s.d.'s in cell parameters are only used when they are defined by crystal symmetry. An approximate (isotropic) treatment of cell e.s.d.'s is used for estimating e.s.d.'s involving l.s. planes.
Refinement. Refinement of *F*^2^ against ALL reflections. The weighted *R*-factor *wR* and goodness of fit *S* are based on *F*^2^, conventional *R*-factors *R* are based on *F*, with *F* set to zero for negative *F*^2^. The threshold expression of *F*^2^ > σ(*F*^2^) is used only for calculating *R*-factors(gt) *etc*. and is not relevant to the choice of reflections for refinement. *R*-factors based on *F*^2^ are statistically about twice as large as those based on *F*, and *R*- factors based on ALL data will be even larger.

### Fractional atomic coordinates and isotropic or equivalent isotropic displacement parameters (Å^2^)

**Table:**

	*x*	*y*	*z*	*U*_iso_*/*U*_eq_	
C1	0.0187 (3)	0.4802 (3)	0.7889 (2)	0.0854 (10)	
H1A	0.0726	0.5120	0.8044	0.128*	
H1B	−0.0388	0.4759	0.8316	0.128*	
H1C	−0.0252	0.5354	0.7376	0.128*	
C2	0.1669 (2)	0.3475 (2)	0.71499 (16)	0.0515 (6)	
C3	0.2258 (2)	0.2272 (2)	0.70449 (18)	0.0602 (7)	
H3	0.2079	0.1622	0.7386	0.072*	
C4	0.3105 (2)	0.2030 (2)	0.64426 (17)	0.0571 (7)	
H4	0.3494	0.1219	0.6378	0.069*	
C5	0.3380 (2)	0.2985 (2)	0.59338 (15)	0.0442 (5)	
C6	0.2771 (2)	0.4189 (2)	0.60244 (16)	0.0548 (6)	
H6	0.2939	0.4841	0.5676	0.066*	
C7	0.1915 (2)	0.4437 (2)	0.66262 (17)	0.0563 (7)	
H7	0.1505	0.5254	0.6678	0.068*	
C8	0.5125 (2)	0.1538 (2)	0.52980 (15)	0.0486 (6)	
H8	0.5188	0.0776	0.5659	0.058*	
C9	0.5849 (2)	0.1689 (2)	0.46494 (14)	0.0442 (5)	
C10	0.5441 (2)	0.2991 (2)	0.42581 (14)	0.0446 (5)	
C11	0.4482 (2)	0.3611 (2)	0.46830 (15)	0.0476 (6)	
H11	0.4041	0.4480	0.4564	0.057*	
C12	0.7299 (2)	0.1887 (2)	0.34512 (14)	0.0430 (5)	
C13	0.8184 (2)	0.1662 (2)	0.29066 (15)	0.0444 (5)	
C14	0.9907 (2)	0.0657 (2)	0.19511 (15)	0.0469 (6)	
C15	1.0237 (3)	−0.1466 (2)	0.1501 (2)	0.0684 (8)	
H15A	1.0782	−0.2149	0.1275	0.082*	
H15B	1.0068	−0.1837	0.2063	0.082*	
C16	0.9051 (3)	−0.0780 (3)	0.09887 (18)	0.0773 (9)	
H16A	0.8485	−0.0134	0.1226	0.116*	
H16B	0.8705	−0.1364	0.0981	0.116*	
H16C	0.9205	−0.0405	0.0431	0.116*	
C17	0.9521 (2)	0.1906 (2)	0.16305 (15)	0.0485 (6)	
C18	1.1509 (3)	0.2438 (3)	0.0997 (2)	0.0904 (12)	
H18A	1.1488	0.2678	0.1506	0.109*	
H18B	1.2049	0.1551	0.1099	0.109*	
C19	1.1992 (3)	0.3184 (4)	0.0333 (3)	0.1182 (16)	
H19A	1.2185	0.2824	−0.0130	0.177*	
H19B	1.2721	0.3178	0.0538	0.177*	
H19C	1.1384	0.4035	0.0158	0.177*	
C20	−0.5385 (3)	0.8748 (3)	0.7996 (2)	0.0770 (9)	
H20A	−0.5821	0.8834	0.7511	0.116*	
H20B	−0.5959	0.9151	0.8368	0.116*	
H20C	−0.4941	0.7871	0.8270	0.116*	
C21	−0.3663 (2)	0.8889 (2)	0.72209 (15)	0.0460 (5)	
C22	−0.3512 (2)	0.7916 (2)	0.68927 (16)	0.0537 (6)	
H22	−0.4039	0.7510	0.7029	0.064*	
C23	−0.2572 (2)	0.7543 (2)	0.63580 (17)	0.0535 (6)	
H23	−0.2467	0.6880	0.6142	0.064*	
C24	−0.17888 (19)	0.8141 (2)	0.61409 (13)	0.0402 (5)	
C25	−0.1954 (2)	0.9128 (2)	0.64690 (15)	0.0469 (5)	
H25	−0.1438	0.9546	0.6325	0.056*	
C26	−0.2882 (2)	0.9490 (2)	0.70089 (16)	0.0517 (6)	
H26	−0.2983	1.0146	0.7232	0.062*	
C27	−0.0765 (2)	0.7044 (2)	0.50572 (14)	0.0450 (5)	
H27	−0.1322	0.6706	0.5018	0.054*	
C28	0.0251 (2)	0.6924 (2)	0.46045 (14)	0.0424 (5)	
C29	0.0835 (2)	0.7582 (2)	0.48538 (14)	0.0414 (5)	
C30	0.0163 (2)	0.8089 (2)	0.54558 (15)	0.0448 (5)	
H30	0.0336	0.8573	0.5731	0.054*	
C31	0.21143 (19)	0.6709 (2)	0.36860 (14)	0.0403 (5)	
C32	0.2935 (2)	0.6418 (2)	0.31059 (14)	0.0408 (5)	
C33	0.4041 (2)	0.5729 (2)	0.18181 (14)	0.0435 (5)	
C34	0.3035 (2)	0.5950 (3)	0.03313 (16)	0.0587 (7)	
H34A	0.3066	0.5558	−0.0107	0.070*	
H34B	0.2317	0.5971	0.0648	0.070*	
C35	0.2904 (3)	0.7273 (3)	−0.00531 (19)	0.0732 (9)	
H35A	0.2825	0.7683	0.0376	0.110*	
H35B	0.2185	0.7724	−0.0419	0.110*	
H35C	0.3616	0.7259	−0.0362	0.110*	
C36	0.4630 (2)	0.6343 (2)	0.20262 (15)	0.0450 (5)	
C37	0.5531 (3)	0.8156 (3)	0.1410 (2)	0.0684 (8)	
H37A	0.5403	0.8307	0.1955	0.082*	
H37B	0.6228	0.8334	0.1184	0.082*	
C38	0.4416 (3)	0.9048 (3)	0.0860 (2)	0.0906 (11)	
H38A	0.4501	0.8856	0.0334	0.136*	
H38B	0.4331	0.9896	0.0776	0.136*	
H38C	0.3701	0.8964	0.1118	0.136*	
N1	0.42782 (17)	0.27241 (16)	0.53236 (12)	0.0454 (5)	
N2	−0.08223 (16)	0.77573 (17)	0.55855 (11)	0.0421 (4)	
O1	0.08803 (17)	0.35935 (18)	0.77861 (12)	0.0664 (5)	
O2	−0.45483 (16)	0.93211 (17)	0.77612 (12)	0.0638 (5)	
S1	0.71479 (6)	0.06700 (5)	0.42617 (4)	0.05735 (19)	
S2	0.62565 (6)	0.34433 (6)	0.34207 (4)	0.05800 (19)	
S3	0.92856 (6)	0.01481 (5)	0.29013 (4)	0.05201 (17)	
S4	0.84103 (7)	0.28873 (6)	0.21607 (4)	0.05770 (18)	
S5	1.10304 (6)	−0.04916 (7)	0.15474 (5)	0.0687 (2)	
S6	0.99851 (6)	0.26916 (7)	0.07115 (4)	0.06066 (19)	
S7	0.09330 (6)	0.61643 (7)	0.38385 (4)	0.05587 (17)	
S8	0.21920 (5)	0.75478 (6)	0.43717 (4)	0.04944 (16)	
S9	0.29160 (6)	0.54706 (6)	0.24866 (4)	0.05071 (16)	
S10	0.42090 (5)	0.68105 (6)	0.29521 (4)	0.05067 (16)	
S11	0.43992 (6)	0.49999 (6)	0.10086 (4)	0.05701 (18)	
S12	0.59037 (6)	0.65155 (7)	0.15249 (5)	0.06202 (19)	

### Atomic displacement parameters (Å^2^)

**Table:**

	*U*^11^	*U*^22^	*U*^33^	*U*^12^	*U*^13^	*U*^23^
C1	0.097 (2)	0.081 (2)	0.105 (3)	−0.0512 (19)	0.042 (2)	−0.051 (2)
C2	0.0554 (14)	0.0509 (13)	0.0568 (15)	−0.0296 (12)	0.0023 (12)	−0.0155 (12)
C3	0.0663 (16)	0.0521 (14)	0.0682 (18)	−0.0355 (13)	0.0141 (13)	−0.0110 (13)
C4	0.0640 (15)	0.0417 (13)	0.0671 (17)	−0.0263 (12)	0.0062 (13)	−0.0106 (12)
C5	0.0444 (12)	0.0428 (12)	0.0471 (13)	−0.0218 (10)	−0.0045 (10)	−0.0069 (10)
C6	0.0663 (16)	0.0430 (13)	0.0565 (16)	−0.0290 (12)	0.0068 (12)	−0.0063 (11)
C7	0.0628 (15)	0.0441 (13)	0.0652 (17)	−0.0250 (12)	0.0078 (13)	−0.0163 (12)
C8	0.0563 (14)	0.0358 (11)	0.0489 (14)	−0.0186 (11)	−0.0008 (11)	−0.0040 (10)
C9	0.0487 (12)	0.0357 (11)	0.0456 (13)	−0.0168 (10)	−0.0052 (10)	−0.0059 (10)
C10	0.0519 (13)	0.0376 (11)	0.0430 (13)	−0.0217 (10)	−0.0054 (10)	−0.0017 (10)
C11	0.0511 (13)	0.0357 (11)	0.0506 (14)	−0.0172 (10)	−0.0017 (11)	−0.0034 (10)
C12	0.0507 (12)	0.0396 (11)	0.0398 (12)	−0.0202 (10)	−0.0027 (10)	−0.0083 (9)
C13	0.0537 (13)	0.0408 (11)	0.0429 (13)	−0.0234 (10)	−0.0028 (10)	−0.0102 (10)
C14	0.0464 (12)	0.0482 (13)	0.0483 (14)	−0.0248 (11)	0.0054 (10)	−0.0088 (11)
C15	0.0791 (18)	0.0409 (13)	0.081 (2)	−0.0226 (13)	0.0314 (16)	−0.0208 (13)
C16	0.108 (2)	0.079 (2)	0.0578 (19)	−0.0528 (19)	0.0120 (17)	−0.0171 (16)
C17	0.0498 (13)	0.0501 (13)	0.0482 (14)	−0.0272 (11)	0.0007 (10)	−0.0064 (11)
C18	0.0609 (18)	0.090 (2)	0.104 (3)	−0.0400 (17)	−0.0002 (17)	0.012 (2)
C19	0.091 (2)	0.121 (3)	0.145 (4)	−0.071 (2)	0.028 (2)	−0.001 (3)
C20	0.0716 (18)	0.076 (2)	0.090 (2)	−0.0347 (16)	0.0337 (17)	−0.0331 (18)
C21	0.0497 (13)	0.0392 (11)	0.0452 (13)	−0.0149 (10)	0.0014 (10)	−0.0109 (10)
C22	0.0571 (14)	0.0534 (14)	0.0618 (17)	−0.0319 (12)	0.0115 (12)	−0.0207 (12)
C23	0.0632 (15)	0.0511 (13)	0.0640 (17)	−0.0353 (12)	0.0140 (12)	−0.0270 (12)
C24	0.0464 (12)	0.0398 (11)	0.0371 (12)	−0.0201 (10)	−0.0018 (9)	−0.0096 (9)
C25	0.0566 (13)	0.0463 (12)	0.0486 (14)	−0.0304 (11)	0.0012 (11)	−0.0143 (11)
C26	0.0631 (15)	0.0442 (12)	0.0562 (16)	−0.0250 (12)	0.0028 (12)	−0.0218 (11)
C27	0.0539 (13)	0.0515 (13)	0.0463 (13)	−0.0345 (11)	0.0056 (10)	−0.0188 (11)
C28	0.0517 (13)	0.0451 (12)	0.0401 (12)	−0.0282 (11)	0.0034 (10)	−0.0136 (10)
C29	0.0483 (12)	0.0445 (12)	0.0380 (12)	−0.0263 (10)	−0.0039 (9)	−0.0079 (10)
C30	0.0511 (13)	0.0500 (13)	0.0487 (14)	−0.0316 (11)	0.0014 (10)	−0.0192 (11)
C31	0.0440 (12)	0.0385 (11)	0.0403 (12)	−0.0202 (10)	−0.0037 (9)	−0.0068 (9)
C32	0.0454 (12)	0.0389 (11)	0.0388 (12)	−0.0190 (10)	−0.0021 (9)	−0.0078 (9)
C33	0.0435 (12)	0.0371 (11)	0.0421 (13)	−0.0107 (10)	0.0009 (9)	−0.0076 (9)
C34	0.0603 (15)	0.0762 (18)	0.0533 (16)	−0.0344 (14)	0.0063 (12)	−0.0293 (14)
C35	0.0787 (19)	0.0650 (18)	0.0669 (19)	−0.0230 (16)	−0.0143 (15)	−0.0098 (15)
C36	0.0445 (12)	0.0421 (12)	0.0453 (13)	−0.0165 (10)	0.0035 (10)	−0.0095 (10)
C37	0.0676 (17)	0.0814 (19)	0.081 (2)	−0.0502 (16)	0.0237 (15)	−0.0345 (17)
C38	0.095 (2)	0.0674 (19)	0.109 (3)	−0.0432 (18)	0.013 (2)	−0.0102 (19)
N1	0.0498 (11)	0.0365 (9)	0.0453 (11)	−0.0181 (9)	−0.0031 (9)	−0.0024 (8)
N2	0.0490 (10)	0.0440 (10)	0.0418 (11)	−0.0258 (9)	0.0026 (8)	−0.0139 (8)
O1	0.0771 (12)	0.0638 (11)	0.0718 (13)	−0.0420 (10)	0.0255 (10)	−0.0241 (10)
O2	0.0664 (11)	0.0601 (11)	0.0746 (13)	−0.0291 (9)	0.0227 (10)	−0.0336 (10)
S1	0.0687 (4)	0.0355 (3)	0.0552 (4)	−0.0152 (3)	0.0100 (3)	−0.0049 (3)
S2	0.0700 (4)	0.0366 (3)	0.0557 (4)	−0.0186 (3)	0.0126 (3)	−0.0027 (3)
S3	0.0593 (4)	0.0405 (3)	0.0521 (4)	−0.0215 (3)	0.0061 (3)	−0.0057 (3)
S4	0.0749 (4)	0.0411 (3)	0.0591 (4)	−0.0287 (3)	0.0121 (3)	−0.0113 (3)
S5	0.0528 (4)	0.0558 (4)	0.0887 (6)	−0.0200 (3)	0.0244 (4)	−0.0157 (4)
S6	0.0604 (4)	0.0621 (4)	0.0538 (4)	−0.0315 (3)	0.0010 (3)	0.0035 (3)
S7	0.0670 (4)	0.0736 (4)	0.0572 (4)	−0.0491 (4)	0.0194 (3)	−0.0360 (3)
S8	0.0544 (3)	0.0618 (4)	0.0499 (4)	−0.0379 (3)	0.0061 (3)	−0.0210 (3)
S9	0.0609 (4)	0.0521 (3)	0.0512 (4)	−0.0329 (3)	0.0092 (3)	−0.0189 (3)
S10	0.0519 (3)	0.0606 (4)	0.0514 (4)	−0.0319 (3)	0.0066 (3)	−0.0203 (3)
S11	0.0609 (4)	0.0493 (3)	0.0573 (4)	−0.0143 (3)	0.0073 (3)	−0.0237 (3)
S12	0.0493 (3)	0.0661 (4)	0.0737 (5)	−0.0261 (3)	0.0183 (3)	−0.0237 (4)

### Geometric parameters (Å, °)

**Table:**

C1—O1	1.404 (3)	C20—O2	1.416 (3)
C1—H1A	0.9600	C20—H20A	0.9600
C1—H1B	0.9600	C20—H20B	0.9600
C1—H1C	0.9600	C20—H20C	0.9600
C2—O1	1.365 (3)	C21—O2	1.368 (3)
C2—C7	1.377 (4)	C21—C22	1.376 (3)
C2—C3	1.386 (4)	C21—C26	1.379 (3)
C3—C4	1.375 (4)	C22—C23	1.385 (3)
C3—H3	0.9300	C22—H22	0.9300
C4—C5	1.379 (3)	C23—C24	1.379 (3)
C4—H4	0.9300	C23—H23	0.9300
C5—C6	1.380 (3)	C24—C25	1.387 (3)
C5—N1	1.424 (3)	C24—N2	1.431 (3)
C6—C7	1.382 (4)	C25—C26	1.380 (3)
C6—H6	0.9300	C25—H25	0.9300
C7—H7	0.9300	C26—H26	0.9300
C8—C9	1.357 (3)	C27—C28	1.359 (3)
C8—N1	1.383 (3)	C27—N2	1.379 (3)
C8—H8	0.9300	C27—H27	0.9300
C9—C10	1.413 (3)	C28—C29	1.413 (3)
C9—S1	1.748 (2)	C28—S7	1.740 (2)
C10—C11	1.357 (3)	C29—C30	1.360 (3)
C10—S2	1.741 (2)	C29—S8	1.747 (2)
C11—N1	1.381 (3)	C30—N2	1.381 (3)
C11—H11	0.9300	C30—H30	0.9300
C12—C13	1.333 (3)	C31—C32	1.338 (3)
C12—S2	1.766 (2)	C31—S8	1.763 (2)
C12—S1	1.768 (2)	C31—S7	1.769 (2)
C13—S4	1.759 (2)	C32—S9	1.756 (2)
C13—S3	1.761 (2)	C32—S10	1.759 (2)
C14—C17	1.341 (3)	C33—C36	1.339 (3)
C14—S5	1.741 (3)	C33—S11	1.751 (2)
C14—S3	1.767 (2)	C33—S9	1.758 (2)
C15—C16	1.504 (4)	C34—C35	1.498 (4)
C15—S5	1.816 (3)	C34—S11	1.812 (3)
C15—H15A	0.9700	C34—H34A	0.9700
C15—H15B	0.9700	C34—H34B	0.9700
C16—H16A	0.9600	C35—H35A	0.9600
C16—H16B	0.9600	C35—H35B	0.9600
C16—H16C	0.9600	C35—H35C	0.9600
C17—S4	1.749 (3)	C36—S12	1.744 (2)
C17—S6	1.756 (2)	C36—S10	1.764 (3)
C18—C19	1.471 (4)	C37—C38	1.497 (4)
C18—S6	1.801 (3)	C37—S12	1.803 (3)
C18—H18A	0.9700	C37—H37A	0.9700
C18—H18B	0.9700	C37—H37B	0.9700
C19—H19A	0.9600	C38—H38A	0.9600
C19—H19B	0.9600	C38—H38B	0.9600
C19—H19C	0.9600	C38—H38C	0.9600
			
O1—C1—H1A	109.5	C21—C22—H22	120.2
O1—C1—H1B	109.5	C23—C22—H22	120.2
H1A—C1—H1B	109.5	C24—C23—C22	121.1 (2)
O1—C1—H1C	109.5	C24—C23—H23	119.5
H1A—C1—H1C	109.5	C22—C23—H23	119.5
H1B—C1—H1C	109.5	C23—C24—C25	118.9 (2)
O1—C2—C7	125.3 (2)	C23—C24—N2	120.9 (2)
O1—C2—C3	115.5 (2)	C25—C24—N2	120.2 (2)
C7—C2—C3	119.1 (2)	C26—C25—C24	120.0 (2)
C4—C3—C2	120.7 (2)	C26—C25—H25	120.0
C4—C3—H3	119.6	C24—C25—H25	120.0
C2—C3—H3	119.6	C21—C26—C25	120.7 (2)
C3—C4—C5	120.2 (2)	C21—C26—H26	119.6
C3—C4—H4	119.9	C25—C26—H26	119.6
C5—C4—H4	119.9	C28—C27—N2	107.7 (2)
C4—C5—C6	119.1 (2)	C28—C27—H27	126.2
C4—C5—N1	120.0 (2)	N2—C27—H27	126.2
C6—C5—N1	120.9 (2)	C27—C28—C29	108.0 (2)
C5—C6—C7	120.9 (2)	C27—C28—S7	134.67 (19)
C5—C6—H6	119.6	C29—C28—S7	117.37 (18)
C7—C6—H6	119.6	C30—C29—C28	107.7 (2)
C2—C7—C6	119.9 (2)	C30—C29—S8	135.34 (19)
C2—C7—H7	120.0	C28—C29—S8	116.94 (18)
C6—C7—H7	120.0	C29—C30—N2	107.7 (2)
C9—C8—N1	107.5 (2)	C29—C30—H30	126.1
C9—C8—H8	126.2	N2—C30—H30	126.1
N1—C8—H8	126.2	C32—C31—S8	123.12 (18)
C8—C9—C10	108.2 (2)	C32—C31—S7	120.17 (19)
C8—C9—S1	134.61 (18)	S8—C31—S7	116.59 (13)
C10—C9—S1	117.11 (18)	C31—C32—S9	122.41 (18)
C11—C10—C9	107.6 (2)	C31—C32—S10	123.97 (19)
C11—C10—S2	134.99 (18)	S9—C32—S10	113.36 (13)
C9—C10—S2	117.30 (18)	C36—C33—S11	126.19 (19)
C10—C11—N1	107.9 (2)	C36—C33—S9	117.20 (19)
C10—C11—H11	126.0	S11—C33—S9	116.18 (15)
N1—C11—H11	126.0	C35—C34—S11	112.9 (2)
C13—C12—S2	120.85 (18)	C35—C34—H34A	109.0
C13—C12—S1	122.40 (18)	S11—C34—H34A	109.0
S2—C12—S1	116.69 (13)	C35—C34—H34B	109.0
C12—C13—S4	121.99 (18)	S11—C34—H34B	109.0
C12—C13—S3	124.17 (18)	H34A—C34—H34B	107.8
S4—C13—S3	113.72 (14)	C34—C35—H35A	109.5
C17—C14—S5	125.62 (19)	C34—C35—H35B	109.5
C17—C14—S3	116.62 (19)	H35A—C35—H35B	109.5
S5—C14—S3	117.63 (14)	C34—C35—H35C	109.5
C16—C15—S5	114.7 (2)	H35A—C35—H35C	109.5
C16—C15—H15A	108.6	H35B—C35—H35C	109.5
S5—C15—H15A	108.6	C33—C36—S12	124.2 (2)
C16—C15—H15B	108.6	C33—C36—S10	116.78 (18)
S5—C15—H15B	108.6	S12—C36—S10	118.41 (15)
H15A—C15—H15B	107.6	C38—C37—S12	114.2 (2)
C15—C16—H16A	109.5	C38—C37—H37A	108.7
C15—C16—H16B	109.5	S12—C37—H37A	108.7
H16A—C16—H16B	109.5	C38—C37—H37B	108.7
C15—C16—H16C	109.5	S12—C37—H37B	108.7
H16A—C16—H16C	109.5	H37A—C37—H37B	107.6
H16B—C16—H16C	109.5	C37—C38—H38A	109.5
C14—C17—S4	117.72 (19)	C37—C38—H38B	109.5
C14—C17—S6	127.2 (2)	H38A—C38—H38B	109.5
S4—C17—S6	115.09 (14)	C37—C38—H38C	109.5
C19—C18—S6	110.6 (3)	H38A—C38—H38C	109.5
C19—C18—H18A	109.5	H38B—C38—H38C	109.5
S6—C18—H18A	109.5	C11—N1—C8	108.78 (19)
C19—C18—H18B	109.5	C11—N1—C5	125.79 (19)
S6—C18—H18B	109.5	C8—N1—C5	125.38 (19)
H18A—C18—H18B	108.1	C27—N2—C30	108.90 (19)
C18—C19—H19A	109.5	C27—N2—C24	124.90 (19)
C18—C19—H19B	109.5	C30—N2—C24	126.1 (2)
H19A—C19—H19B	109.5	C2—O1—C1	118.4 (2)
C18—C19—H19C	109.5	C21—O2—C20	118.4 (2)
H19A—C19—H19C	109.5	C9—S1—C12	94.27 (11)
H19B—C19—H19C	109.5	C10—S2—C12	94.50 (11)
O2—C20—H20A	109.5	C13—S3—C14	95.12 (12)
O2—C20—H20B	109.5	C17—S4—C13	95.40 (11)
H20A—C20—H20B	109.5	C14—S5—C15	100.98 (12)
O2—C20—H20C	109.5	C17—S6—C18	102.71 (14)
H20A—C20—H20C	109.5	C28—S7—C31	94.32 (11)
H20B—C20—H20C	109.5	C29—S8—C31	94.39 (11)
O2—C21—C22	124.5 (2)	C32—S9—C33	95.30 (12)
O2—C21—C26	115.8 (2)	C32—S10—C36	95.20 (12)
C22—C21—C26	119.7 (2)	C33—S11—C34	101.04 (12)
C21—C22—C23	119.7 (2)	C36—S12—C37	101.95 (12)
